# The dimerisable Cre recombinase allows conditional genome editing in the mosquito stages of *Plasmodium berghei*

**DOI:** 10.1371/journal.pone.0236616

**Published:** 2020-10-12

**Authors:** Priyanka Fernandes, Sylvie Briquet, Delphine Patarot, Manon Loubens, Bénédicte Hoareau-Coudert, Olivier Silvie

**Affiliations:** 1 Centre d’Immunologie et des Maladies Infectieuses, INSERM, CNRS, CIMI-Paris, Sorbonne Université, Paris, France; 2 UMS PASS, Plateforme de Cytométrie de la Pitié-Salpêtrière (CyPS), Sorbonne Université, Paris, France; Bernhard Nocht Institute for Tropical Medicine, GERMANY

## Abstract

Asexual blood stages of the malaria parasite are readily amenable to genetic modification via homologous recombination, allowing functional studies of parasite genes that are not essential in this part of the life cycle. However, conventional reverse genetics cannot be applied for the functional analysis of genes that are essential during asexual blood-stage replication. Various strategies have been developed for conditional mutagenesis of *Plasmodium*, including recombinase-based gene deletion, regulatable promoters, and mRNA or protein destabilization systems. Among these, the dimerisable Cre (DiCre) recombinase system has emerged as a powerful approach for conditional gene deletion in *P*. *falciparum*. In this system, the bacteriophage Cre is expressed in the form of two separate, enzymatically inactive polypeptides, each fused to a different rapamycin-binding protein. Rapamycin-induced heterodimerization of the two components restores recombinase activity. We have implemented the DiCre system in the rodent malaria parasite *P*. *berghei*, and show that rapamycin-induced excision of floxed DNA sequences can be achieved with very high efficiency in both mammalian and mosquito parasite stages. This tool can be used to investigate the function of essential genes not only in asexual blood stages, but also in other parts of the malaria parasite life cycle.

## Introduction

The life cycle of *Plasmodium* parasites is complex, and involves several stages of development and differentiation within the vertebrate host and *Anopheles* vector. In mammals, infection begins when motile forms of the parasite known as sporozoites are transmitted by infected *Anopheles* mosquitoes to the host. These sporozoites first go through an obligatory intra-hepatic step, where they multiply into thousands of merozoites that are released into the blood stream, where they begin the next round of replication within erythrocytes. Some merozoites develop into gametocytes which fuse to form a motile ookinete in the gut of the mosquito. These ookinetes encyst on the basal side of the midgut wall, where they further differentiate into sporozoites that invade the salivary glands, and are transmitted by the mosquito during the next blood-meal [1].

Experimental genetics have been instrumental in deciphering key aspects of the parasite biology. However, a thorough understanding of the genes that are critical for several stages of development, is lacking. The *Plasmodium* genome is haploid during the intra-erythrocytic stage of development and is thus easily amenable to genetic modification via homologous recombination. Hence most gene modification systems are adapted to targeting genes at the blood-stage of the parasite life cycle. However, as conventional reverse genetic strategies cannot be used to target genes that are essential for blood-stage replication, the functional analysis of these genes in other stages of development remains largely unexplored.

Various strategies have previously been developed for conditional mutagenesis in *Plasmodium*, including recombinase-based gene deletion [2], regulatable promoters [3], promoter swap strategies with known [4–7] or synthetic promoters [8], and mRNA or protein destabilization systems [9–12]. While most of these strategies are stage-specific, others require a promoter that is expressed by distinct stages of the parasite, thus limiting the investigation of essential genes in multiple developmental stages. In recent years, the dimerisable Cre (DiCre) recombinase system has emerged as an efficient system for conditional gene knockout in *P*. *falciparum* and other apicomplexans [2, 13]. It consists of the expression of the bacteriophage Cre in the form of two separate, enzymatically inactive polypeptides, each fused to a different rapamycin-binding protein (either FKBP12 or FRB) that heterodimerise after the addition of rapamycin [14]. Heterodimerisation of the two components in turn restores recombinase activity, leading to site-specific excision of Lox-flanked DNA sequences and rapid and efficient rapamycin-induced gene deletion [2, 13].

DiCre-expressing parasite lines have previously been generated in both *P*. *falciparum* [2, 15] and *P*. *berghei* [16] and used to investigate the function of genes in asexual or sexual blood stages of development. The introduction of lox sites inside artificial silent introns (LoxPint) further improved the methodology by permitting the floxing of genomic regions without affecting protein expression [17]. A more recent study developed a DiCre-expressing *P*. *falciparum* parasite line in an attempt to investigate the function of AMA1 in mosquito stages [18]. Here, using a similar approach, we investigated whether the DiCre system can be used for conditional gene manipulation in the insect stages of the rodent parasite *P*. *berghei*. We generated a *P*. *berghei* parasite line that stably expresses both components of the DiCre cassette, along with a fluorescent marker to facilitate the phenotyping of excised and non-excised parasites in all developmental stages. After validating that the resulting parasite line showed no phenotypical defect as compared to the parental strain, we show that a single rapamycin dose administered to mice is sufficient to induce efficient excision of a floxed target DNA sequence *in vivo* in mice, including in transmission stages. In addition, we adapted the rapamycin treatment to mosquitoes, and show that Cre-mediated excision can be used to delete genes in mosquito stages, thus demonstrating the versatility and robustness of the DiCre system for conditional genome editing across the parasite life cycle.

## Materials and methods

### Ethics statement

All animal work was conducted in strict accordance with the Directive 2010/63/EU of the European Parliament and Council on the protection of animals used for scientific purposes. Protocols were approved by the Ethical Committee Charles Darwin N°005 (approval #7475–2016110315516522).

### Generation of plasmids for transfection

#### DiCre plasmid

The DiCre plasmid was designed to replace the GFP cassette previously introduced in the *p230p* locus of *P*. *berghei* ANKA parasites [19] by mCherry, DiCre and *T*. *gondii* dihydrofolate reductase/thymidylate synthase (TgDHFR/TS) cassettes. The DiCre plasmid was generated by assembling five DNA fragments: a mCherry cassette flanked by a 800-kb 3’ fragment of *P*. *berghei* HSP70 promoter and PbDHFR 3’UTR (DC1); a Cre59 coding sequence followed by PfCAM 3’UTR (DC2); a bidirectional eEF1α promoter (DC3); a Cre60 coding sequence followed by the 3’ UTR of PbHSP70 (DC4); and a sequence corresponding to the 3’ UTR of PbDHFR (DC5). All elements of the DiCre plasmid were amplified by PCR using standard PCR conditions (using the CloneAmp HiFi PCR premix) and sequentially ligated (In-Fusion HD Cloning Kit, Clontech) into an acceptor plasmid containing a TgDHFR/TS cassette flanked by two LoxP sites. The DC2 and DC4 fragments were amplified using genomic DNA from DiCre-expressing *P*. *falciparum* parasites (kind gift of Kai Wengelnik and Mike Blackman). The resulting plasmid sequence was verified by Sanger sequencing (Eurofins Genomics) and linearized with *Sca*I and *Dra*III before transfection. The DC1 and DC5 fragments served as homology regions for double homologous recombination between the HSP70 promoter the PbDHFR 3’ UTR sequences flanking GFP at the modified p230p locus of PbGFP parasites. All primers used to construct the DiCre plasmid are listed in S1 Table in S1 File.

#### PbDCIII plasmid

The PbDCIII plasmid was designed to replace the mCherry cassette of the DiCre parental line with a cassette containing two fluorescent markers, GFP and ECFP, separated by a silent intron taken from the *P*. *berghei slarp* gene (PBANKA_0902100) [20] and containing an inserted LoxN site [17]. To permit the selection of transfected parasites, we also included a human dihydrofolate reductase marker (hDHFR) downstream of the GFP cassette, followed by the 3’ UTR of *P*. *berghei* calmodulin (CAM) gene. A 2A skip peptide was introduced between GFP and hDHFR to enable transcription of both cassettes under the control of a single HSP70 promoter. In this construct, the GFP-2A-hDHFR-PbCAM cassette was flanked by two silent introns containing LoxN sites. The LoxN-GFP-2A-hDHFR and LoxN-ECFP fragments were ordered as synthetic genes (Eurofins Genomics). All elements of the PbDCIII plasmid were amplified by PCR using standard PCR conditions (using the CloneAmp HiFi PCR premix) and sequentially ligated into the *Sph*I-*Sal*I restriction sites of a pUC18 vector (In-Fusion HD Cloning Kit, Clontech). The resulting plasmid sequence was verified by Sanger sequencing (Eurofins Genomics) and linearized with *Nar*I before transfection. All primers used to construct the GFP-ECFP plasmid are listed in S1 Table in S1 File.

### Experimental animals, parasites and cell lines

Female SWISS mice (6–8 weeks old, from Janvier Labs) were used for all routine parasite infections. Parasite lines were maintained in mice through intraperitoneal injections of frozen parasite stocks and transmitted to *Anopheles stephensi* mosquitoes for experimental purposes. *P*. *berghei* sporozoites were isolated from infected female *Anopheles* mosquitoes and intravenously injected into C57BL/6J mice (female, 4–6 weeks old, Janvier Labs). A drop of blood from the tail was collected in 1ml PBS daily and used to monitor the parasitaemia by flow cytometry.

For parasite transfection, schizonts purified from an overnight culture of PbGFP or PbDiCre parasites were transfected with 5–10 μg of linearized plasmid by electroporation using the AMAXA Nucleofector device (program U033), as previously described [21], and immediately injected intravenously into the tail vein of SWISS mice. To permit the selection of resistant transgenic parasites, pyrimethamine (35 mg/L) was added to the drinking water and administered to mice, one day after transfection. The parasitaemia was monitored daily by flow cytometry and the mice sacrificed at a parasitaemia of 2–3%. The mice were bled and the infected blood collected for preparation of frozen stocks and isolation of parasites for genomic DNA extraction.

Pure transgenic parasite populations were isolated by flow cytometry-assisted sorting of mCherry or GFP-expressing blood stage parasites on a FACSAria II (Becton-Dickinson), as described [19]. Mice were injected intraperitoneally with frozen parasite stocks and monitored until the parasitaemia was between 0.1 and 1%. On the day of sorting, one drop of blood from the tail was collected in 1 ml PBS and used for sorting of 100 pRBCs which were recovered in 200 μl RPMI +20% foetal calf serum (FCS), and then injected intravenously into two mice (100 μl each). The mice were sacrificed at a parasitaemia of 2–3% and the blood recovered for preparation of frozen stocks and genotyping.

*Anopheles stephensi* mosquitoes were reared at 24–26°C with 80% humidity and permitted to feed on infected mice that were anaesthetised, using standard methods of mosquito infection as previously described [22]. Post-feeding, *P*. *berghei*-infected mosquitoes were kept at 21°C and fed on a 10% sucrose solution. Salivary gland sporozoites were collected between 21 and 28 days post-feeding from infected mosquitoes, by hand dissection and homogenisation of isolated salivary glands in complete DMEM (DMEM supplemented with 10% FCS, 1% Penicillin-Streptomycin and 1% L-Glutamine). Mosquitoes infected with GFP-expressing parasites were sorted under a fluorescence microscope prior to dissection.

HepG2 cells (ATCC HB-8065) were cultured in DMEM supplemented with 10% FCS, 1% Penicillin-Streptomycin and 1% L-Glutamine as previously described [23].

### Rapamycin treatment

DiCre recombinase mediated excision of targeted DNA sequences *in vivo* was achieved by administration of 200 μg Rapamycin (1mg/ml stock, Rapamune, Pfizer) to mice by oral gavage, 24 hours prior to transmission to mosquitoes. In order to achieve excision in the mosquito stages, 10 μg rapamycin (1 mg/ml stock solution in DMSO, Sigma-Aldrich) was added to 10 ml 10% sucrose solution and used to feed mosquitoes. The rapamycin dose was refreshed every alternate day along with the sucrose solution.

### Genotyping PCR

Infected mice were sacrificed at a parasitaemia of 2–3% and the infected blood collected and passed through a CF11 column (Whatman) to deplete leucocytes. The RBCs collected were then centrifuged and lysed with 0.2% saponin (Sigma) to recover parasite material for genomic DNA isolation using a kit (Qiagen DNA Easy Blood and Tissue Kit), according to the manufacturer’s instructions. Specific PCR primers were designed to check for wild-type and recombined loci and are listed in S2 Table in S1 File. All PCR reactions were carried out using Recombinant Taq DNA Polymerase (5U/μl from Thermo Scientific) and standard PCR cycling conditions.

### *In vitro* infections, immunofluorescence assays and microscopy

#### Infection of hepatocytes

HepG2 cells were seeded in collagen-coated culture plates, at a density of 30,000 cells/well in a 96-well plate for flow cytometry analysis or 100,000 cells/well in 8 well μ-slide (IBIDI) for immunofluorescence assays, 24 hours prior to infection with sporozoites. On the day of infection, the culture medium in the wells was refreshed with complete DMEM, followed by the addition of 10,000 sporozoites and incubation for 3 hours at 37°C. After 3 hours, the wells were washed twice with complete DMEM and then incubated for another 24–48 hours at 37°C and 5% CO_2_.

For quantification of EEF numbers, the cells were trypsinized after two washes with PBS, followed by addition of complete DMEM and one round of centrifugation. After discarding the supernatant, the cells were either directly re-suspended in FACS buffer (PBS + 3% FCS) for flow cytometry, or fixed with 2% PFA for 10 minutes, subsequently washed once with PBS and then re-suspended in PBS. Cells were then analyzed on a Guava EasyCyte 6/2L bench cytometer equipped with 488 nm and 532 nm lasers (Millipore).

#### Immunofluorescence assays

For immunofluorescence assays, the cells were washed twice with PBS, then fixed with 4% PFA for 10 minutes followed by two washes with PBS, quenching with glycine 0,1 M for 5 minutes, permeabilization with 1% Triton X-100 for 5 minutes before washes with PBS and blocking in PBS with 3% bovine serum albumin (BSA). Cells were then incubated for 1h with goat anti-UIS4 primary antibody (1:500, Sicgen), followed by donkey anti-goat Alexa Fluor 594 or Alexa Fluor 488 secondary antibody (1:1000, Life Technologies).

#### Fluorescence microscopy

Live samples such as blood-stages, midguts and salivary glands from infected mosquitoes, were mounted in PBS and visualised live using a fluorescence microscope (Zeiss Axio Observer.Z1 fluorescence microscope equipped with a LD Plan-Neofluar 403/0.6 Corr Ph2 M27 objective). Due to spectral overlap between GFP and CFP channels, we set the exposure time according to the positive control and maintained the same exposure for both excised and non-excised parasites, in order to allow comparisons. All images were processed with ImageJ for adjustment of contrast.

## Results

### Generation of a DiCre-expressing *P*. *berghei* line

**Fig 1:**
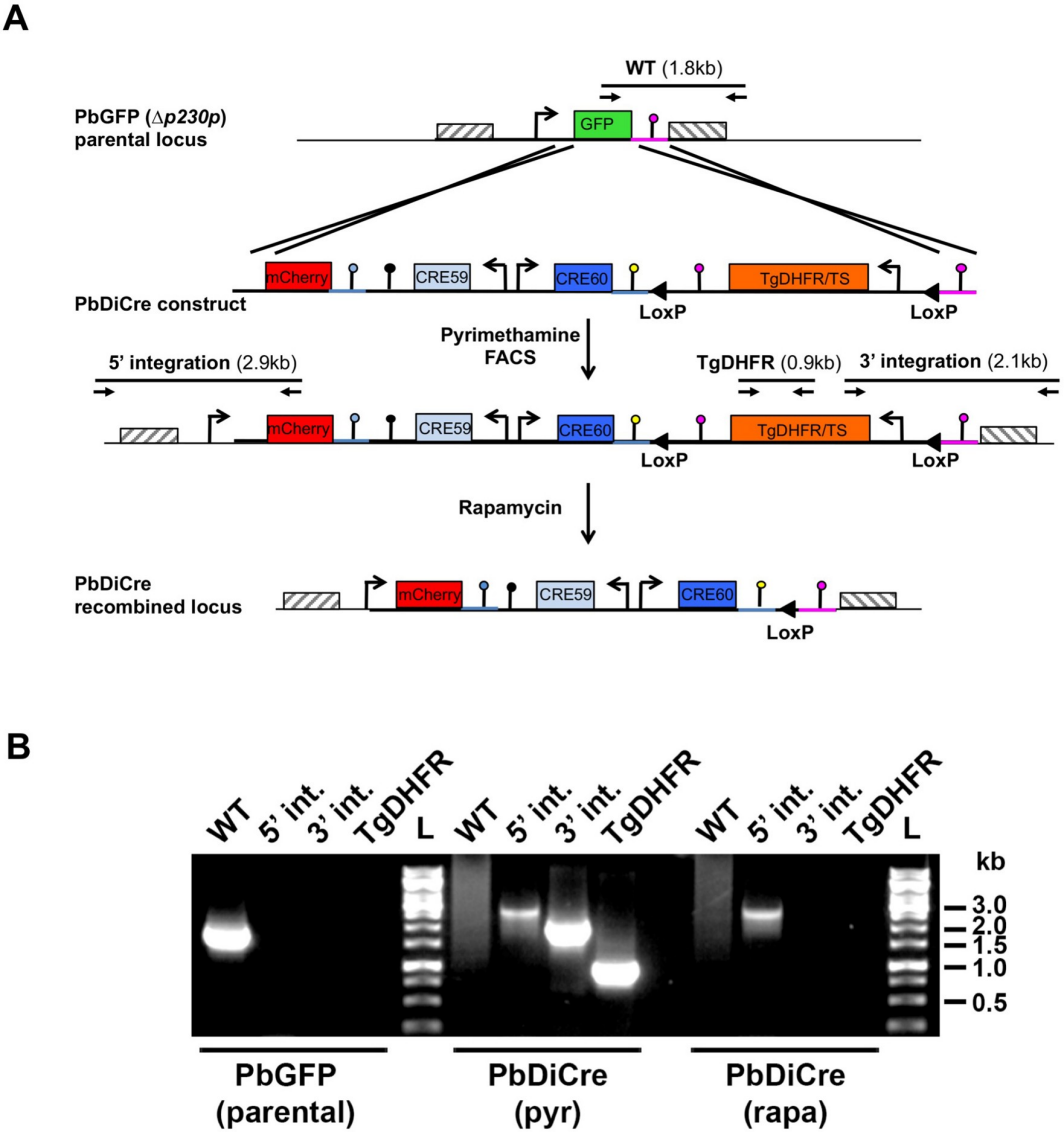
Generation of DiCre-expressing *P*. *berghei* parasites. **A.** Replacement strategy to modify the PbGFP locus for DiCre expression. The DiCre plasmid, containing the two Cre components in addition to a mCherry cassette and a TgDHFR/TS pyrimethamine resistance cassette, was integrated into a pre-modified P230p locus in *P*.*berghei*, where the P230p gene was previously replaced by a GFP cassette [19]. Following parasite transfection and selection with pyrimethamine, mCherry+ parasites were sorted by flow cytometry to exclude any residual GFP+ population. Genotyping primers and expected PCR fragments are indicated by arrows and lines, respectively. **B.** PCR analysis of genomic DNA isolated from parental PbGFP, pyrimethamine-selected (pyr) and rapamycin-treated (rapa) PbDiCre parasites. Confirmation of the predicted recombination events was achieved with primer combinations specific for WT, 5’ or 3’ integration. The TgDHFR primer combination was used to confirm the loss of the TgDHFR/TS cassette following rapamycin treatment.

We first generated a parasite line that stably and constitutively expresses both components of the Cre enzyme. We assembled a construct encoding the N-terminal Cre 59 (residues Thr19-Asn59) and C-terminal Cre 60 (Asn60-Asp343) portions of the Cre fused at their N-terminus to FKBP12 and FRB, respectively (Fig 1A). The two components were placed under control of the constitutive bidirectional promoter eEF1alpha, and followed by the 3’ untranslated region (UTR) from PfCAM and PbHSP70, respectively. In addition, the construct contained a mCherry cassette under the control of an inactive truncated fragment of HSP70 promoter, and a TgDHFR/TS pyrimethamine resistance cassette. The TgDHFR/TS cassette was flanked by two LoxP sites, to allow Cre-mediated excision and production of drug selectable marker-free parasites (Fig 1A). A sequence corresponding to the 3’ UTR of PbDHFR was included at the end of the construct, for homologous recombination at the modified *p230p* locus of GFP-expressing *P*. *berghei* ANKA parasites (PbGFP) [19] (Fig 1A).

Following transfection of PbGFP parasites, integration of the construct by double homologous recombination resulted in the replacement of GFP by mCherry and reconstitution of a functional HSP70 promoter to drive mCherry expression, along with insertion of the DiCre and TgDHFR/TS cassettes. Transfected parasites were selected with pyrimethamine and mCherry-positive parasites were sorted by FACS. The resulting parasite population was exposed to a single dose of rapamycin that was administered orally to mice. This treatment resulted in excision of the TgDHFR/TS cassette, as demonstrated by PCR genotyping (Fig 1B), and retention of a single LoxP site. Cloning by limiting dilution resulted in the final selectable-marker free mCherry-expressing PbDiCre parasite line.

### PbDiCre parasites progress normally across the parasite life cycle

**Fig 2:**
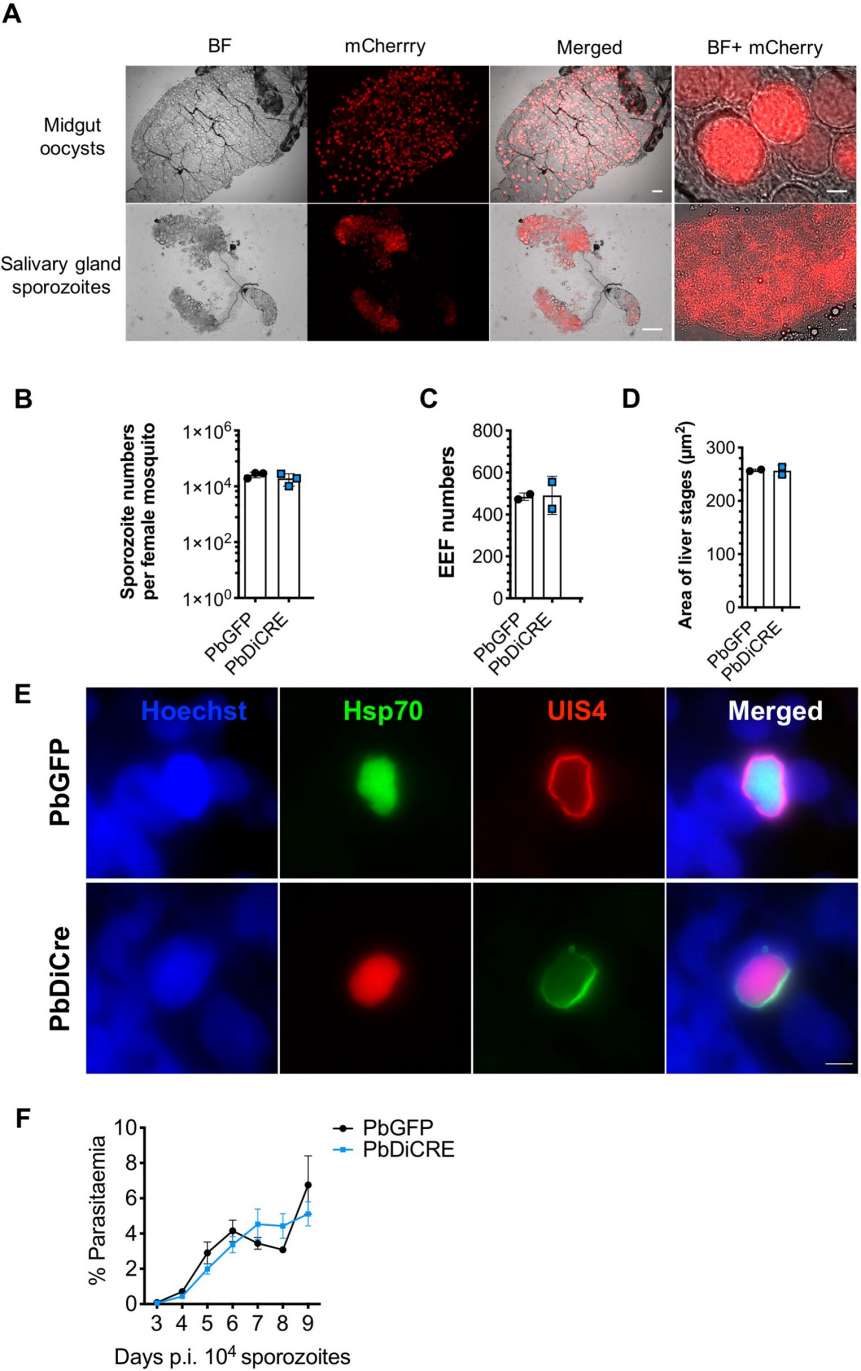
PbDiCre parasites show no defect in mosquito and liver stages. **A.** Fluorescence microscopy imaging of unfixed midgut oocysts and salivary glands from PbDiCre-infected mosquitoes. Scale bar, 100 μm for the midguts and salivary glands, and 10 μm for the magnified images. **B.** Comparison of sporozoite numbers isolated from salivary glands of female mosquitoes infected with PbGFP or PbDiCre parasites. Data shown are mean +/- SD of three independent experiments. **C.** Quantification of EEFs in HepG2 cell cultures infected with PbGFP or PbDiCre sporozoites. Data shown are mean +/- SD of two independent experiments. **D.** Quantification of EEF size (area) in HepG2 cell cultures infected with PbGFP or PbDiCre sporozoites. Data shown are mean +/- SD of two independent experiments. **E.** Images of PbGFP and PbDiCre EEFs in HepG2 infected cell cultures, 48 hours post-infection. Scale bar, 10 μm. **F.** Comparison of parasite development *in vivo* post-sporozoite injection. C57BL6/J mice (n = 5) were injected intravenously with 1 x 10^4^ PbGFP or PbDiCre sporozoites. Parasitemia was then followed daily by FACS. The data shown are mean +/- SEM of 5 mice per group.

To exclude any interference of the DiCre cassette with parasite life cycle progression, we examined if these parasites could be transmitted to mosquitoes and back to mice. PbDiCre parasites formed oocysts in the midgut of infected mosquitoes and developed into sporozoites that colonized the insect salivary glands (Fig 2A). Similar numbers of salivary gland sporozoites were recovered from mosquitoes infected with PbDiCre and parental PbGFP parasites (Fig 2B), confirming that PbDiCre parasites develop normally in the mosquito. In addition, when incubated with a monolayer of HepG2 cells, PbDiCre sporozoites formed similar numbers of exo-erythrocytic forms (EEFs) as PbGFP parasites (Fig 2C). Liver stage development of PbDiCre parasites was comparable to PbGFP, as evidenced by EEF size (Fig 2D) and formation of a UIS4-labeled PVM (Fig 2E). Finally, we injected PbDiCre and PbGFP sporozoites into C57BL/6J mice and observed no difference in either pre-patency or blood-stage parasitaemia between PbGFP- and PbDiCre-infected mice (Fig 2F), showing that PbDiCre parasites are capable of completing the parasite life cycle, similarly to the parental PbGFP parasites.

In summary, these results confirmed that parasites constitutively expressing the DiCre cassette progress normally through the parasite life cycle, and can thus be used as a parental strain to target genes of interest.

### Exposure to rapamycin during the mammalian blood stages allows efficient recovery of recombined mosquito stages

**Fig 3:**
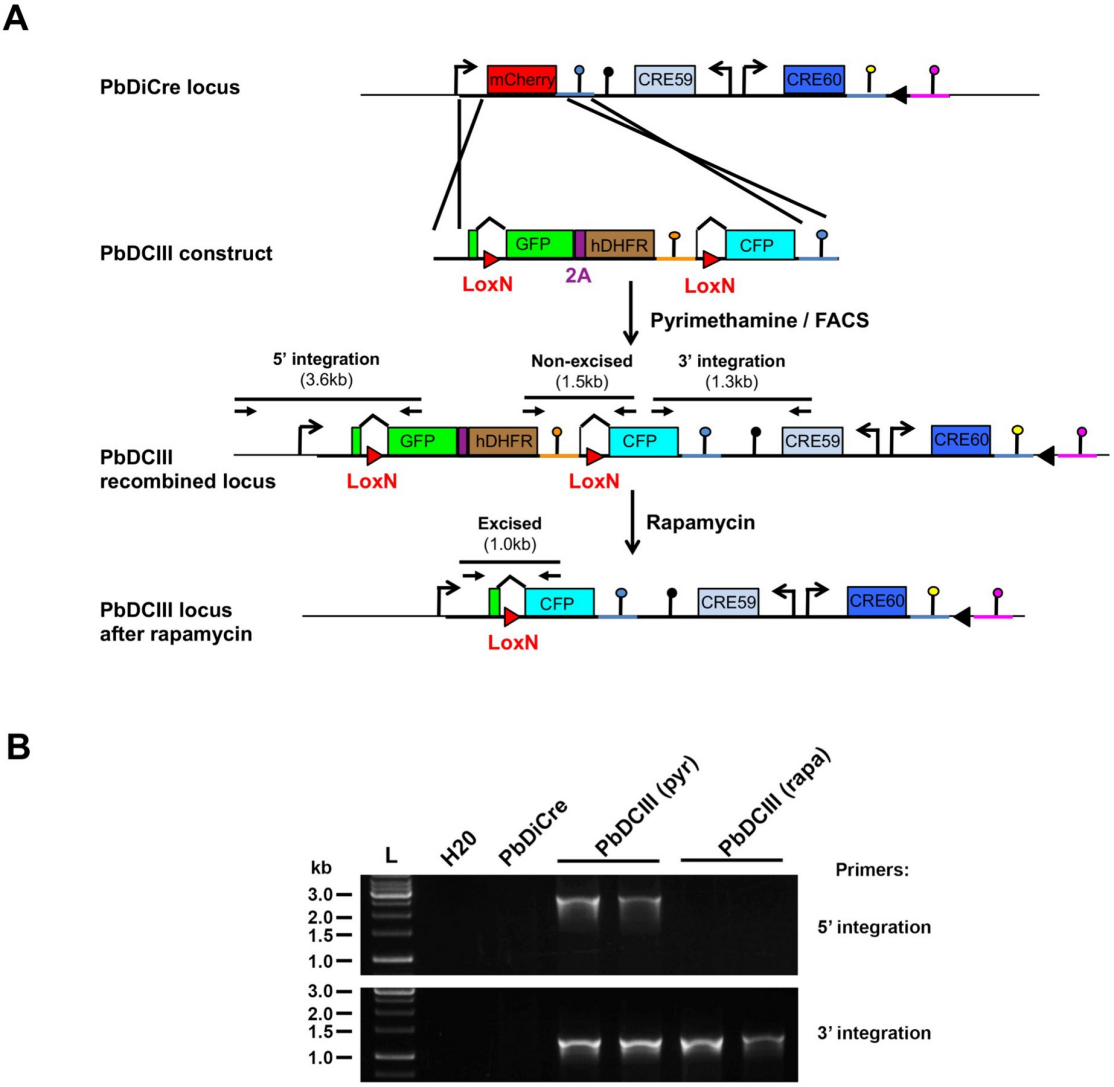
Generation of a PbDCIII reporter line to monitor Cre activity. **A**. Replacement strategy to modify the mCherry locus in PbDiCre parasites and generate the PbDCIII parasite line. The DCIII construct contains a GFP-hDHFR cassette flanked by LoxN sites, and followed by a CFP cassette. Integration of the construct results in GFP expression instead of mCherry. Following rapamycin treatment, Cre-mediated recombination results in excision of the GFP cassette and expression of CFP. Genotyping primers and expected PCR fragments are indicated by arrows and lines, respectively. **B.** PCR analysis of genomic DNA isolated from parental PbDiCre, pyrimethamine-selected (pyr) and rapamycin-treated (rapa) PbDCIII parasites. Confirmation of the predicted recombination events was achieved with primer combinations specific for 5’ and 3’ integration.

In order to assess the efficacy and robustness of the DiCre system to target genes of interest during parasite transmission to the mosquito, we designed a reporter construct that allows a simple assessment of site-specific rapamycin-induced DNA excision, using fluorescent markers. The designed construct replaced the endogenous mCherry cassette in PbDiCre parasites with a gene sequence comprised of GFP-2A-hDHFR-PbCAMutr flanked by LoxN sites, and placed under the inactive truncated fragment of HSP70 promoter (Fig 3A). We selected LoxN sites which are not compatible with LoxP sites, in order to avoid recombination with the single LoxP site, retained after excision of the TgDHFR/TS cassette in the PbDiCre parasite line. An additional eCFP cassette lacking a promoter was inserted downstream of the second LoxN site (Fig 3A) thereby permitting selective expression of GFP or CFP before or after rapamycin-induced excision, respectively. Proper integration of the construct was confirmed by genotyping PCR (Fig 3B), resulting in a PbDCIII parasite line that provided us with a suitable tool to visually monitor rapamycin-induced excision in all developmental stages of the parasite.

**Fig 4:**
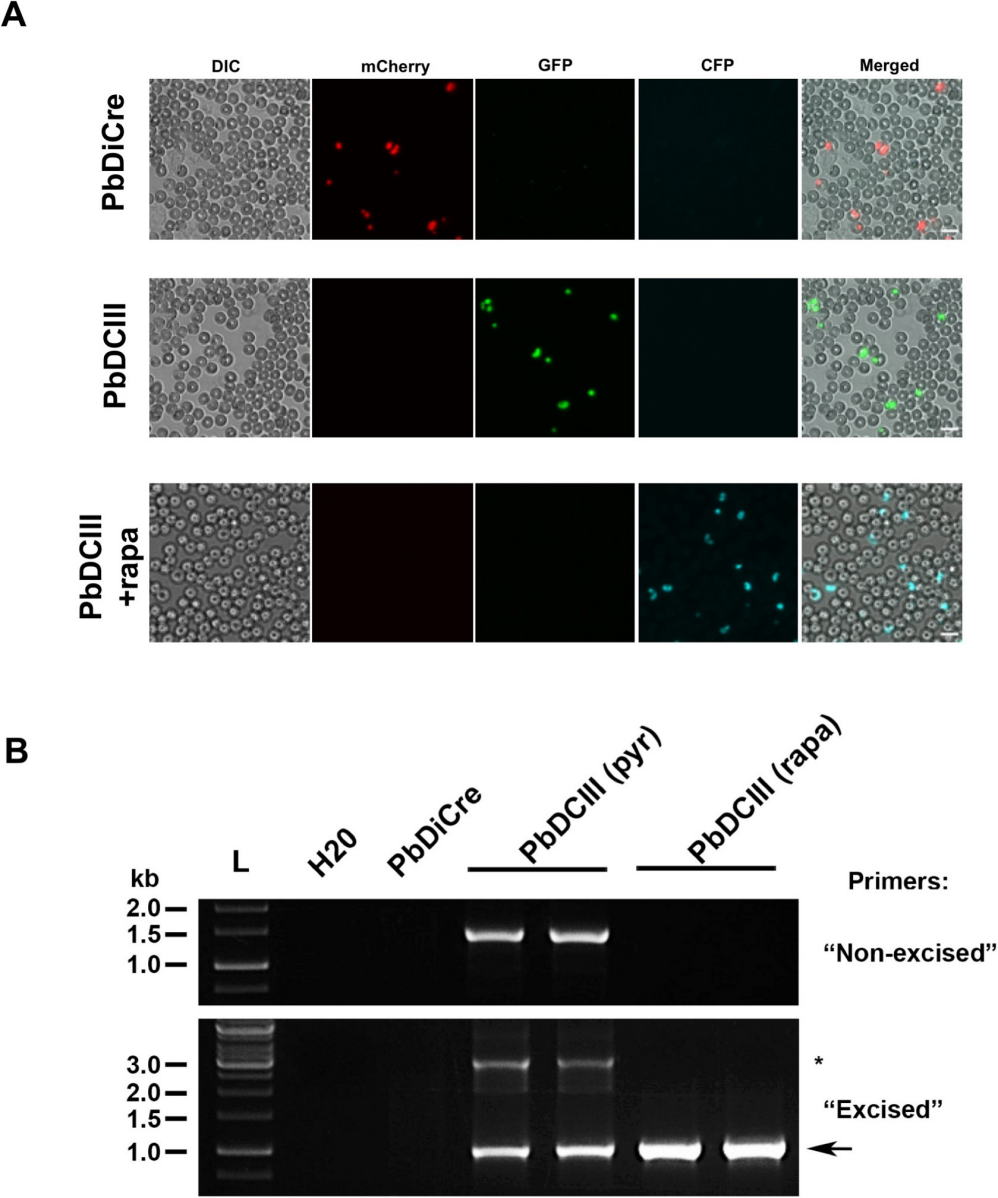
Exposure to rapamycin during the mammalian blood stages. **A.** Fluorescence microscopy of unfixed blood stages in the parental PbDiCre parasites (upper panels) and in PbDCIII parasites before (middle panels) and after (lower panels) rapamycin treatment. Scale bar, 10 μm. **B.** PCR analysis of genomic DNA isolated from parental PbDiCre, pyrimethamine-selected (pyr) and rapamycin-treated (rapa) PbDCIII parasites, using primer combinations specific for non-excised or excised locus. A band corresponding to a non-excised locus can be amplified with the “excised” primer combination and is indicated with an asterisk.

In order to verify that exposure of blood-stages to rapamycin results in efficient gene excision, we infected mice with PbDCIII parasitised red blood cells (pRBCs) and treated them with one dose of rapamycin. Untreated mice were used as controls. At 48h post-treatment, direct examination by fluorescence microscopy confirmed the presence of CFP-positive (CFP+) parasites in the blood of rapamycin-treated mice (Fig 4A), while only GFP-positive (GFP+) parasites were observed in untreated mice, suggesting that gene excision in blood-stages was very efficient after rapamycin treatment. Although genotyping PCR confirmed complete excision in rapamycin-treated PbDCIII parasites, excision bands could also be amplified by PCR in untreated PbDCIII parasites (Fig 4B), suggesting some level of activity of the Cre in the absence of rapamycin.

**Fig 5:**
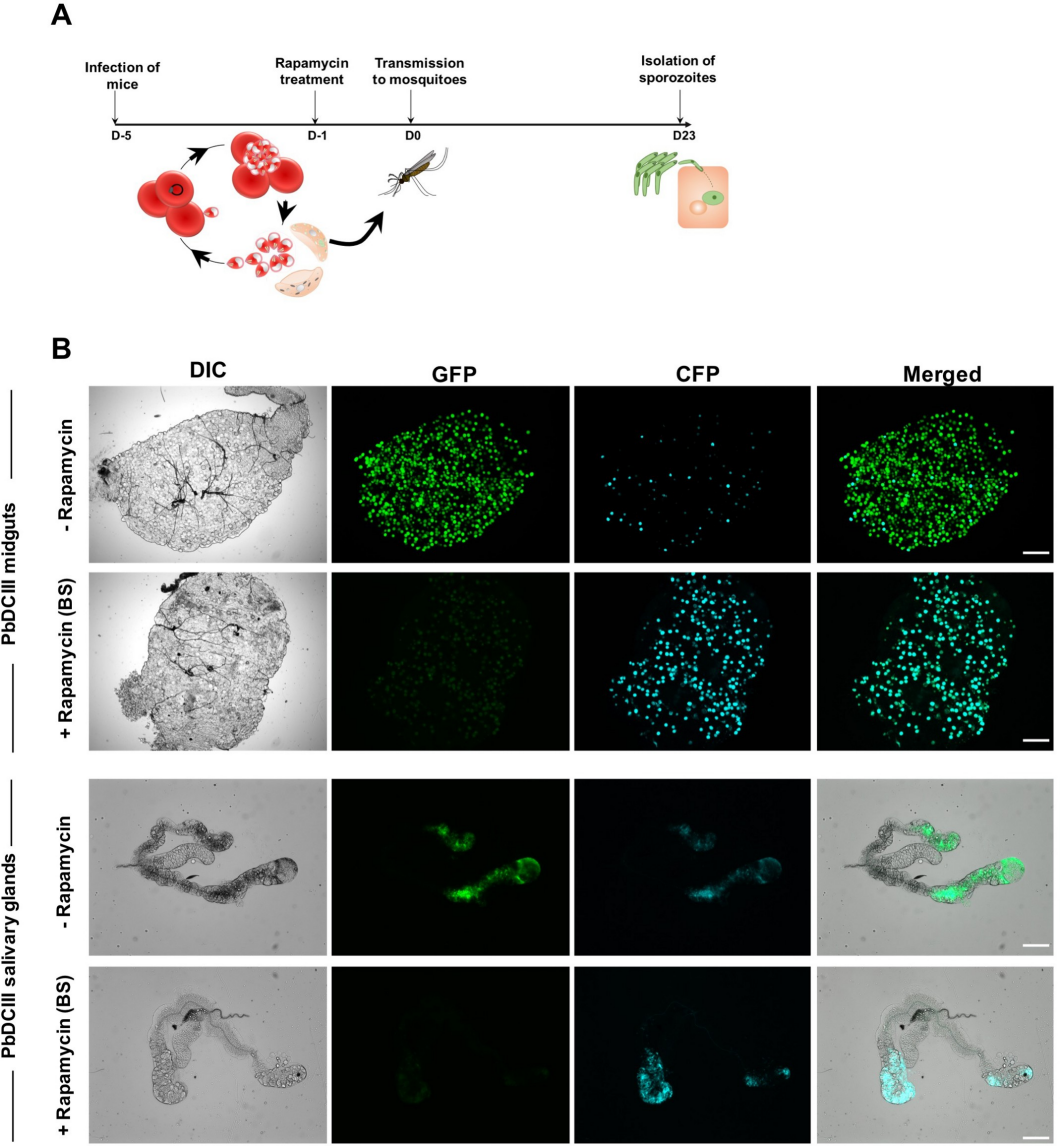
Rapamycin-mediated DNA recombination in sexual blood stages. **A.** Illustration of the rapamycin treatment protocol applied to sexually committed blood stages prior transmission to mosquitoes. **B.** Fluorescence microscopy of unfixed midguts and salivary glands isolated from PbDCIII-infected mosquitoes parasites, before (-rapamycin) or after blood-stage rapamycin treatment (+ rapamycin BS). Scale bar, 200 μm for midguts and 100 μm for the salivary glands.

In the next step, we evaluated if DNA excision could also be induced in sexually committed parasites/gametocytes. After verifying the presence of exflagellating gametocytes in the blood by microscopy, mice infected with PbDCIII parasites were administered rapamycin and then transmitted to mosquitoes, 24 hours post-treatment (Fig 5A). Since gametocyte development takes around 24 hours in *P*. *berghei*, we reasoned that under these conditions all transmitted gametocytes would have undergone excision of the floxed DNA, based on a previous study which established that complete DiCre-mediated recombination can be achieved in less than 24 hours in *P*. *berghei* after rapamycin exposure [16]. Following transmission, infection and development of both treated and untreated parasites in the mosquito was monitored by fluorescence microscopy. We predominantly observed CFP+ oocysts and salivary gland sporozoites in mosquitoes infected with rapamycin-treated PbDCIII parasites, showing that one dose of rapamycin is sufficient to induce efficient excision in transmission stages (Fig 5B).

### Exposure of mosquito stages to rapamycin allows DiCre-mediated gene recombination

**Fig 6:**
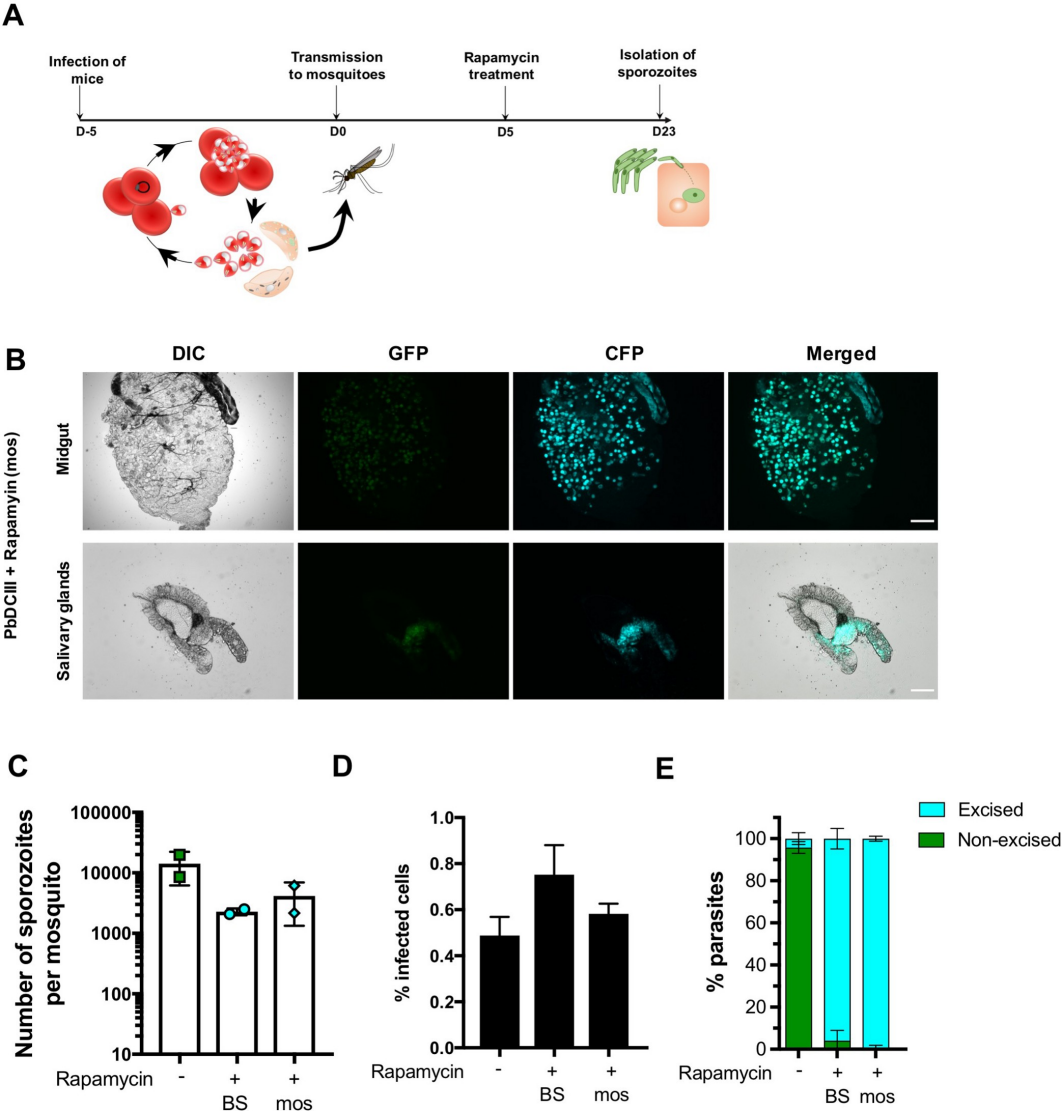
Rapamycin-mediated DNA recombination in mosquito stages. **A.** Illustration of the rapamycin treatment protocol applied to infected mosquitoes. **B.** Fluorescence microscopy of unfixed midguts and salivary glands isolated from PbDCIII-infected rapamycin-treated mosquitoes. Scale bar, 200 μm for midguts, and 100 μm for the salivary glands. **C.** Quantification of salivary gland sporozoites in mosquitoes infected with the PbDCIII parasites, after rapamycin exposure of mice (BS) or mosquitoes (mos) in comparison to the untreated parasites. Results shown are mean+/- SD of two independent experiments. **D.** Quantification of infected cells in HepG2 cell cultures infected with the PbDCIII parasite line, after rapamycin exposure of mice (BS) or mosquitoes (mos) in comparison to untreated control. Results shown are mean+/- SD of five technical replicates and are representative of two independent experiments. **E**. Quantification of excised CFP+ and non-excised GFP+ EEFs *in vitro* in infected HepG2 cell cultures, as determined by FACS. Results shown are mean+/- SD of five technical replicates and are representative of two independent experiments.

Due to optimal activity of the Cre enzyme at 37°C, the DiCre system has only been used so far to manipulate genes in intra-erythrocytic stages of *Plasmodium*. We therefore tested if exposure to rapamycin of infected mosquitoes, instead of blood-stages, would also result in efficient DNA excision. For this purpose, we fed PbDCIII-infected mosquitoes on a sucrose solution containing 1 μg/ml rapamycin, for two weeks, starting at day 5 post-transmission (Fig 6A). Interestingly, both oocysts and salivary glands isolated from treated mosquitoes were CFP+, showing that DiCre-mediated DNA excision can also be induced by treating mosquitoes with rapamycin (Fig 6B).

We then dissected the salivary glands of mosquitoes infected with PbDCIII untreated or rapamycin-treated parasites, either at the blood or mosquito stages, and quantified the number of salivary gland sporozoites. In two independent experiments, rapamycin exposure in mice or mosquitoes was associated with a reduction in the number of sporozoites (Fig 6C). We assume this reduction could in part be due to the sorting of untreated PbDCIII infected mosquitoes, based on GFP fluorescence, prior to dissection. While a similar sorting could not be performed after rapamycin treatment due to excision of the GFP cassette, we cannot exclude, however, a detrimental effect of rapamycin on parasite development in the mosquito. Nevertheless, with both protocols, isolated sporozoites remained infectious to HepG2 cell cultures (Fig 6D), and both rapamycin treatment regimens resulted in almost complete parasite DNA recombination as revealed by the switch from GFP to CFP expression (Fig 6E).

Overall, these results showed that the DiCre system can be used to target genes in different developmental stages both in the mammalian host and in the mosquito vector.

## Discussion

In spite of numerous advances in gene editing technologies that are currently available to investigate the function of *Plasmodium* genes, the role of blood-stage essential genes in mosquito or mammalian pre-erythrocytic developmental stages remains poorly explored. We implemented the DiCre system in *P*. *berghei* parasites and illustrate how the system can be used to conditionally delete genes in *P*. *berghei* mosquito stages. We first generated a marker-free fluorescent parasite line in *P*. *berghei*, which constitutively expresses both components of the dimerisable Cre recombinase [14], by introducing components of the DiCre along with an mCherry cassette into a non-essential P230p locus [24]. Asexual development, sporogony in the mosquito, sporozoite infectivity in mice and EEF development *in vitro* were comparable to a reference PbGFP line [19], showing that the components of the DiCre did not interfere with parasite development or transmission.

We then confirmed the integrity of the Cre components in inducing excision after rapamycin treatment, using the PbDCIII reporter line. A single dose of rapamycin was sufficient to induce near complete excision in blood-stages, as shown by PCR, consistent with previously published data [2, 16]. However, excision events were also noted in untreated PbDCIII parasites by PCR in blood stages and by microscopy in some oocysts and sporozoites. Although these data suggest some leakiness in a minor proportion of parasites, we cannot exclude that co-housing of untreated and rapamycin-treated mice may have resulted in the exposure of untreated mice to rapamycin excreted in the faeces of treated mice. Nevertheless, rapamycin-induced DNA recombination in gametocytes was very efficient as observed by the lack of GFP+ oocysts in the mosquito, suggesting that targeted deletion of blood-stage essential genes could successfully be achieved in sexual stages for functional studies in the mosquito. In particular, the DiCre system is well-suited to the functional investigation of essential genes shared by merozoites and sporozoites, such as *ama1* [18]. Additionally, gene deletion can be achieved in sexual stages, two to three weeks prior to sporozoite isolation, thus limiting the risk of protein lingering after successful gene excision. The DiCre system therefore has an advantage over the previously described Flp/FRT system [25], where gene excision occurs late during sporozoite development, with the risk of incomplete depletion due to protein carryover after DNA recombination [26].

A recent study that generated a *P*. *falciparum* NF54 DiCre parasite line to conditionally knockout *ama1* and *fikk7*.*1* genes, reported a significant reduction in oocyst numbers after transmission of rapamycin treated parasites to mosquitoes [18], for which the authors speculated that treatment with rapamycin was mainly responsible. In agreement with these observations, we detected a reduction in salivary gland sporozoite numbers after rapamycin treatment of PbDCIII parasites. In light of these findings, we sought to circumvent the effects of rapamycin on oocyst formation by treating mosquitoes with rapamycin instead of blood-stages. Mosquitoes infected with untreated PbDCIII parasites were fed with rapamycin 5 days after the blood-meal, to avoid any effects on ookinetes and the formation of oocysts. However, a similar reduction of the number of salivary gland sporozoites was observed irrespective of the rapamycin treatment regimen. In our experiments, the observed reduction in sporozoite numbers might stem in part from the sorting procedure for infected mosquitoes, however we cannot exclude the effects of rapamycin on parasite development in the midgut. This detrimental effect could be due to inhibition of the target of rapamycin (TOR) pathway, which is known to play a role in nutritional sensing in the mosquito [27], and may participate in oocyst formation and/or development. It might therefore be possible to mitigate these effects through titrations in the dose of rapamycin used to treat either the mice or mosquitoes, or by using rapamycin analogues with reduced toxicity, thus leaving room for refinement of the procedure.

We only observed excised CFP+ EEFs in infected hepatocytes confirming that excision of GFP in PbDCIII parasites is efficient after rapamycin treatment of both blood- and mosquito stages. This suggests that rapamycin administration to mosquitoes could be successfully applied to conditionally delete genes involved in gametocytogenesis or ookinete formation and study their function during the subsequent stages of parasite development in the mosquito. Furthermore, rapamycin treatment could also be adapted to different stages in the mosquito, such as treatment of midgut sporozoites prior to salivary gland invasion or treatment of salivary gland sporozoites before hepatocyte invasion, although we predict that the percentage of excision might vary with shorter duration times.

In summary, we generated a fluorescent PbDiCre line and show that it can be used to investigate the function of essential blood-stage genes in mosquito stages. By treating parasites with rapamycin in the blood or in the mosquito, we demonstrate the versatility of the DiCre system in targeting genes of interest at multiple stages of development. Overall, our study introduces a new robust and versatile methodology to conditionally delete genes in different stages of *Plasmodium* parasites, which should help deciphering the function of essential genes across the parasite life cycle.

## Supporting information

S1 File (PDF)

S1 Raw images (PDF)
