# Supplementary material for: The dimerisable Cre recombinase allows conditional genome editing in the mosquito stages of Plasmodium berghei
Source: PLoS One. 2020 Oct 12;15(10):e0236616. doi: 10.1371/journal.pone.0236616 (PMC7549836; doi:10.1371/journal.pone.0236616)
Supplement: S1 File — (PDF) [file pone.0236616.s001.pdf]

Supporting information

The dimerisable Cre recombinase allows conditional genome editing in the mosquito stages of *Plasmodium berghei*

Priyanka Fernandes[1¶], Sylvie Briquet[1¶], Delphine Patarot[1], Manon Loubens[1], Bénédicte Hoareau-Coudert[2], Olivier Silvie[1*]

[1]Sorbonne Université, INSERM, CNRS, Centre d'Immunologie et des Maladies Infectieuses, CIMI-Paris, Paris, France

[2]Sorbonne Université, UMS PASS, Plateforme de Cytométrie de la Pitié-Salpêtrière (CyPS), Paris, France

*Corresponding author

Email: olivier.silvie@inserm.fr

¶These authors contributed equally to the work

Contents:

-S1 Table

-S2 Table

S1 Table. List of primers used to assemble plasmid constructs.

	**Oligonucleotide**	**Sequence 5´ → 3´**
**DiCre construct**	DC1for	AATTGGAGCTCCACCGCGGAGGTATATATAATAGCTGCTTTAACTAAGG
	DC1rev	AGGGCGGCCGCCACCGCGGTCAAGTCCAACTATTTATGAATCATTGAAG
	DC2for	ACGGGATCCAGAATTCGGAACTTAATAAAAAAGAGGAGTAATGTAC
	DC2rev	CATATGAAAATGGCCCCTAAGAAGAAGAGAAAGGTG
	DC3for	GGGGCCATTTTCATATGTATAAAATTTTTATTTATTTATAAGC
	DC3rev	GGGGCCATTTTCATATGTATAAAATTTTTATTTATTTATAAGC
	DC4for	GGCCATTTTCATATGAAAATGGCCCCTAAGAAGAAGAGAAAGGTG
	DC4rev	TAACAGTACAGAATTCTCAGTCCCCATCCTCCAGCAGCCTCACC
	DC5for	GATCGCGGCGCGCCGGTACCGTCTCTTCAATGATTCATAAATAGTTGGAC
	DC5rev	AGGGAACAAAAGCTGGTACCTGAAATTAATAAAATAAAATACATATCCCTCAC
**PbDCIII construct**	HSPpromFor	CCAGTGCCAAGCTTGCATGCAGGTATATATAATAGCTGCTTTAAC
	HSPpromRev	GCTAGCTTTGTAATTGTAATTTATTGGGATAATAATG
	GFP-2A-hDHFRfor	CAATTACAAAGCTAGCATGCTTAAGGCTAGTAAAGGAGAAGAAG
	GFP-2A-hDHFRrev	TTAATCATTCTTCTCATATACTTCAAATTTG
	PbCAMutrFor	GAATGATTAAACTAGTATTATTAATATATATGAATATATATACATCG
	PbCAMutrRev	TATAACATACACTAGTGGAGAGAGAAGAATGTATTTGTGGCACC
	ECFPfor	GAGAAGAATGATTAAACTAGTGTATGTTATATTTATGCATGCCATACTAAC
	ECFPrev	AACGGGATCCGCTAGCTTACTTGTACAGCTCGTCCATGCCG
	PbDHFRutrFor	AATTACAATTACAAAGCTAGCGGATCCCGTTTTTCTTAC
	PbDHFRutrRev	CGGGGATCCTCTAGAGTCGACTTAACTGCAGGATTTTTAAATATGC
	ama1Homology3’Rev	ATTAACTAGTgctagctaaaccccatattatagaaatgcttttcc
**LoxN-GFP-2A-hDHFR synthetic gene**		ATGCTTAAGGCTAGTAAAGGAGAAGAAGTATGTTATATTTATGCATGCCATACT<br>AACATTTGTTGTTTCCATATGAATGCATCATTTACAATAATATATATAACTTCG<br>TATAGCATACATTATACGAAGTTATTTTACATATAAATATTATGAAATTGTATT<br>AATGTAAATTCTTTCCTTCAAAAATACCATGTTAGCTTTTCACTGGAGTTGTCC<br>CAATTCTTGTTGAATTAGATGGTGATGTTAATGGGCACAAATTTTCTGTCAGTG<br>GAGAGGGTGAAGGTGATGCAACATACGGAAAACTTACCCTTAAATTTATTTGCA<br>CTACTGGAAAACTACCTGTTCCATGGCCAACACTTGTCACTACTTTCGCGTATG<br>GTCTTCAATGCTTTGCGAGATACCCAGATCATATGAAACAGCATGACTTTTTCA<br>AGAGTGCCATGCCCGAAGGTTATGTACAGGAAAGAACTATATTTTTCAAAGATG<br>ACGGGAACTACAAGACACGTGCTGAAGTCAAGTTTGAAGGTGATACCCTTGTTA<br>ATAGAATCGAGTTAAAAGGTATTGATTTTAAAGAAGATGGAAACATTCTTGGAC<br>ACAAATTGGAATACAACTATAACTCACACAATGTATACATCATGGCAGACAAAC<br>AAAAGAATGGAATCAAAGTTAACTTCAAAATTAGACACAACATTGAAGATGGAA<br>GCGTTCAACTAGCAGACCATTATCAACAAAATACTCCAATTGGCGATGGCCCTG<br>TCCTTTTACCAGACAACCATTACCTGTCCACACAATCTGCCCTTTCGAAAGATC<br>CCAACGAAAAGAGAGACCACATGGTCCTTCTTGAGTTTGTAACAGCTGCTGGGA<br>TTACACATGGCATGGATGAACTATACAAAGGTTCGGGAGAGGGCAGAGGATCCC<br>TGCTAACATGCGGTGATGTCGAGGAGAATCCTGGCCCAATGGTTGGTTCGCTAA<br>ACTGCATCGTCGCTGTGTCCCAGAACATGGGCATCGGCAAGAACGGGGACCTGC<br>CCTGGCCACCGCTCAGGAACGAATTTAGATATTTCCAGAGAATGACCACAACCT<br>CTTCAGTAGAAGGTAAACAGAATCTGGTGATTATGGGTAAGAAGACCTGGTTCT<br>CCATTCCTGAGAAGAATCGACCTTTAAAGGGTAGAATTAATTTAGTTCTCAGCA<br>GAGAACTCAAGGAACCTCCACAAGGAGCTCATTTTCTTTCCAGAAGTCTAGATG<br>ATGCCTTAAAACTTACTGAACAACCAGAATTAGCAAATAAAGTAGACATGGTCT<br>GGATAGTTGGTGGCAGTTCTGTTTATAAGGAAGCCATGAATCACCCAGGCCATC<br>TTAAACTATTTGTGACAAGGATCATGCAAGACTTTGAAAGTGACACGTTTTTC<br>CAGAAATTGATTTGGAGAAATATAAACTTCTGCCAGAATACCCAGGTGTTCTCT

	CTGATGTCCAGGAGGAGAAAGGCATTAAGTACAAATTTGAAGTATATGAGAAGA<br>ATGATTAA
**LoxN-ECFP synthetic gene**	GTATGTTATATTTATGCATGCCATACTAACATTTGTTGTTTCCATATGAATGCA<br>TCATTTACAATAATAATAACTTCGTATAGCATACATTATACGAAGTTATTATTT<br>TACATATAAATATTATGAAATTGTATTAATGTAAATTCTTTCCTTCAAAAATAC<br>CATGTTAGATGGTGAGCAAGGGCGAGGAGCTGTTCACCGGGGTGGTGCCCATCC<br>TGGTCGAGCTGGACGGCGACGTAAACGGCCACAAGTTCAGCGTGTCCGGCGAGG<br>GCGAGGGCGATGCCACCTACGGCAAGCTGACCCTGAAGTTCATCTGCACCACCG<br>GCAAGCTGCCCGTGCCCTGGCCCACCCTCGTGACCACCCTGACCTGGGGCGTGC<br>AGTGCTTCAGCCGCTACCCCGACCACATGAAGCAGCACGACTTCTTCAAGTCCG<br>CCATGCCCGAAGGCTACGTCCAGGAGCGCACCATCTTCTTCAAGGACGACGGCA<br>ACTACAAGACCCGCGCCGAGGTGAAGTTCGAGGGCGACACCCTGGTGAACCGCA<br>TCGAGCTGAAGGGCATCGACTTCAAGGAGGACGGCAACATCCTGGGGCACAAGC<br>TGGAGTACAACTACATCAGCCACAACGTCTATATCACCGCCGACAAGCAGAAGA<br>ACGGCATCAAGGCCAACTTCAAGATCCGCCACAACATCGAGGACGGCAGCGTGC<br>AGCTCGCCGACCACTACCAGCAGAACACCCCCATCGGCGACGGCCCCGTGCTGC<br>TGCCCGACAACCACTACCTGAGCACCCAGTCCGCCCTGAGCAAAGACCCCAACG<br>AGAAGCGCGATCACATGGTCCTGCTGGAGTTCGTGACCGCCGCCGGGATCACTC<br>TCGGCATGGACGAGCTGTACAAGTAA

S2 Table. List of primers used for parasite genotyping by PCR.

	Oligonucleotide	Sequence 5´ → 3´
**PbDiCRE genotyping**	WTfor	GATGGAAGCGTTCAACTAGCAGACC
	WTrev	AAGTGTTGCAAATATATTACACATGTCATG
	5'intFor	TTTATATGACTTAACGGCAATGCCAAAGG
	5'intRev	TCACCTTCAGCTTGGCGGTCTGG
	3'intFor	ACACGAGAAAAGTGTTTGAAATCTGTGG
	3'intRev	AAGTGTTGCAAATATATTACACATGTCATG
	TgDHFRfor	CAATGAGCTCCTTGACGTCC
	TgDHFRrev	TCGTCGATTTCCTTGATGCG
**PbDCIII genotyping**	5'intFor	TTTATATGACTTAACGGCAATGCCAAAGG
	5'intRev	GCCCATTAACATCACCATCTAATTCAACAAG
	3'intFor	GTCTATATCACCGCCGACAAG
	3'intRev	CTGTGTGCAGATCCTGGGCTGCCTGGTGC
	Non-excisedFor	GAATGATTAAACTAGTATTATTAATATATATGAATATATATACATCG
	Non-excisedRev	GTACAGCTCGTCCATGCCG
	ExcisedFor	CACTATTTTGCCATAAGCAC
	ExcisedRev	GTACAGCTCGTCCATGCCG
