# Supplementary material for: The dimerisable Cre recombinase allows conditional genome editing in the mosquito stages of Plasmodium berghei
Source: PLoS One. 2020 Oct 12;15(10):e0236616. doi: 10.1371/journal.pone.0236616 (PMC7549836; doi:10.1371/journal.pone.0236616)

S1 raw images. Uncropped gel images. **A**. Uncropped gel image corresponding to Fig 1B. **B**. Uncropped gel image corresponding to Fig 3B. **C**. Uncropped gel image corresponding to Fig 4B. The red boxes highlight which parts of the full gel images are reproduced in the corresponding figures.

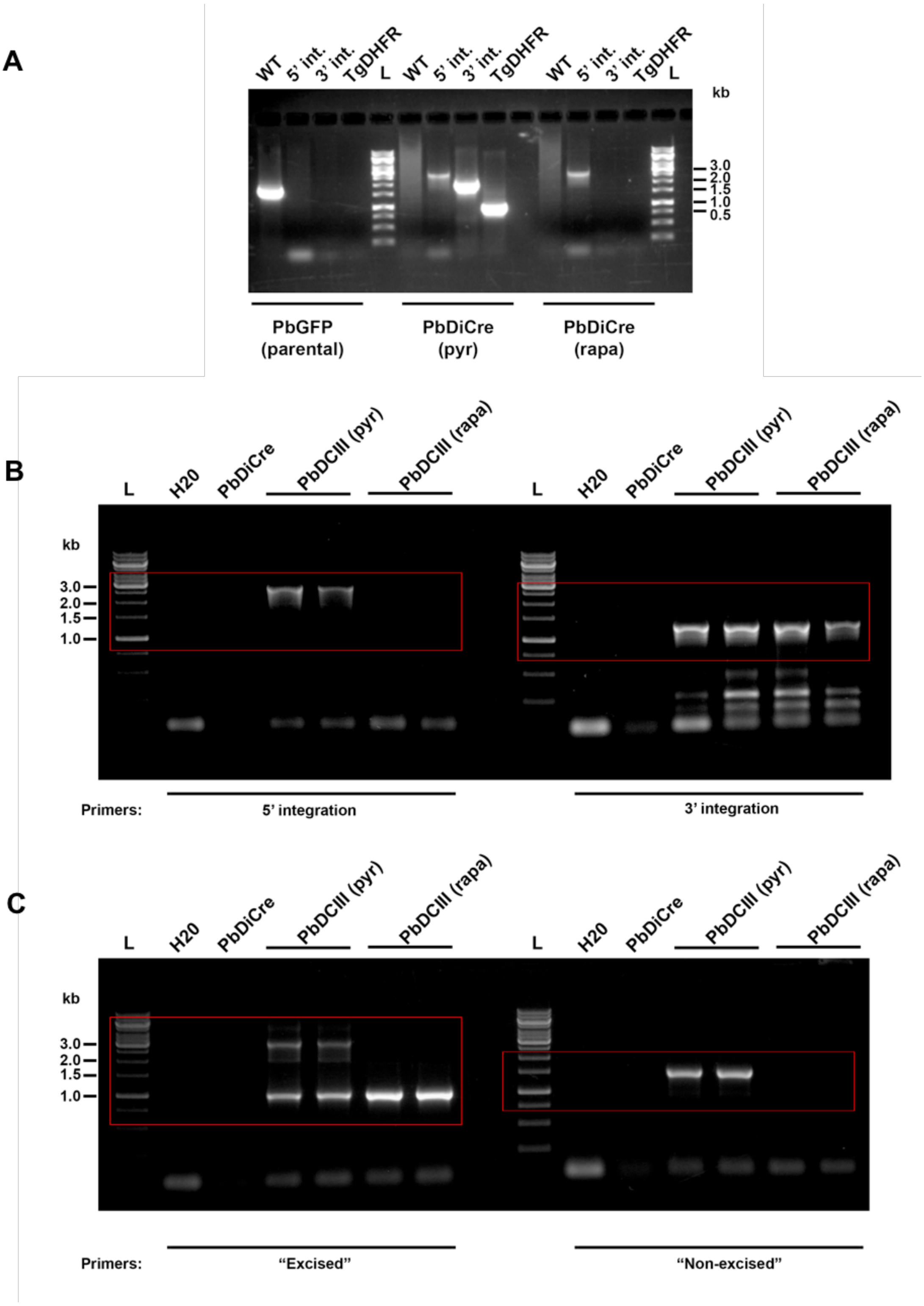

Supplement: S1 Raw images — (PDF) [file pone.0236616.s002.pdf]
